# Supplementary material for: High-resolution spatial mapping of cell state and lineage dynamics in vivo with PEtracer
Source: Science. Author manuscript; Available in PMC 2026 Jan 5. (PMC12766569; doi:10.1126/science.adx3800)
Supplement: adx3800SupplementaryMaterialsresub2v11 [file NIHMS2114502-supplement-adx3800SupplementaryMaterialsresub2v11.docx]

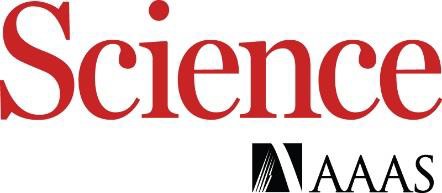


## Supplementary Materials for

**High-resolution spatial mapping of cell state and lineage dynamics in vivo with PEtracer**

**Authors:** Luke W. Koblan^1,2†,*^, Kathryn E. Yost^1,2†,*^, Pu Zheng^1,2†^, William N. Colgan^1,2†^, Matthew G. Jones^3^, Dian Yang^4,5^, Arhan Kumar^1,2^, Jaspreet Sandhu^1,2,6^, Alexandra Schnell^1,2^, Dawei Sun^7,8^, Can Ergen^9,10^, Reuben A. Saunders^1,2,11^, Xiaowei Zhuang^2,12,13,^, William E. Allen^2,11,14^, Nir Yosef^15^, Jonathan S. Weissman^1,2,7*^

### Affiliations:

1Whitehead Institute for Biomedical Research; Cambridge MA, 02142, USA.

2Howard Hughes Medical Institute; Chevy Chase MD, 20815, USA.

3David H. Koch Center for Integrative Cancer Research, Massachusetts Institute of Technology, Cambridge, MA 02139, USA

4Department of Dermatology, Stanford University; Stanford CA, 94063, USA.

5Department of Systems Biology, Columbia University; New York City NY, 10032, USA. 6Department of Molecular Pharmacology and Therapeutics, Columbia University; New York City NY, 10032, USA.

7Division of Gastroenterology, Massachusetts General Hospital; Boston MA, 02114, USA.

8Broad Institute of MIT and Harvard; Cambridge MA, 02142, USA.

9Department of Stem Cell and Regenerative Biology, Harvard University; Cambridge MA, 02138, USA.

10Center for Computational Biology, University of California, Berkeley; Berkeley CA, 94720, USA.

11Department of Electrical Engineering and Computer Sciences, University of California, Berkeley; Berkeley CA, 94720, USA

12Harvard Society of Fellows, Harvard University; Cambridge MA, 02138, USA 13Department of Chemistry and Chemical Biology, Harvard University; Cambridge MA, 02138, USA

14Department of Physics, Harvard University; Cambridge MA, 02138, USA

15Current affiliation: Department of Developmental Biology, Stanford University School of Medicine; Stanford CA, 94305, USA

16Department of Systems Immunology, Weizmann Institute of Science; Rehovot, 7610001, Israel

†These authors contributed equally

*Corresponding authors: [lkoblan@wi.mit.edu](mailto:lkoblan@wi.mit.edu); [kyost@wi.mit.edu](mailto:kyost@wi.mit.edu); [weissman@wi.mit.edu](mailto:weissman@wi.mit.edu)

### The PDF file includes:

Figs. S1 to S16 References

### Other Supplementary Materials for this manuscript include the following:

Tables S1 to S30 Movie S1


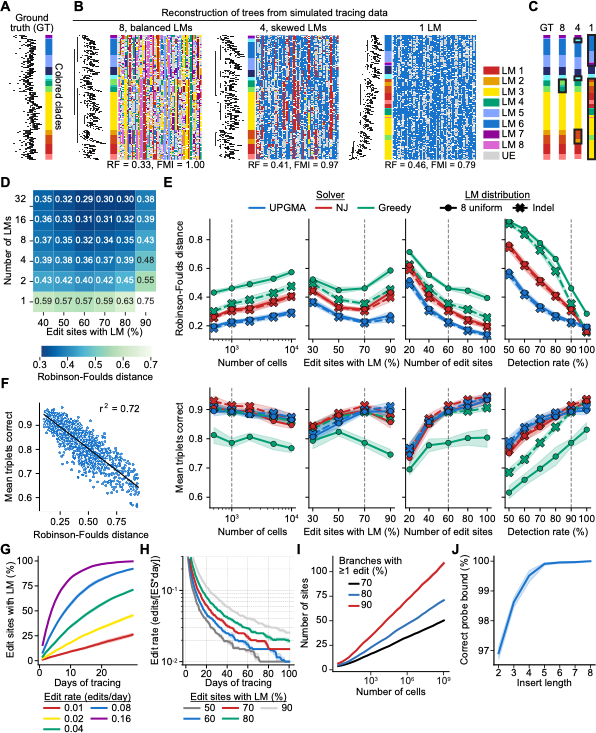


**Fig. S1. *In silico* modeling of key lineage tracing parameters.** (**A**) Simulated ground truth (GT) phylogeny containing 1000 cells. Color bar indicates clade assignment for each cell. (**B**) Trees

reconstructed using neighbor joining for simulated tracing data with eight balanced LMs (left), four skewed LMs (center), or one LM (left). Robinson-Foulds (RF) reconstruction error relative to GT tree. Color bars indicate clade in GT tree and clade consistency quantified using Fowlkes- Mallow Index (FMI), a precision-recall metric. (**C**) Comparison of clade structure between trees with black outlines indicating discrepancies relative to GT tree. (**D**) Mean Robinson-Foulds reconstruction error for simulated phylogenies varying the number of lineage marks (LMs) and fraction of edit sites with an LM installed. (**E**) Mean Robinson-Foulds reconstruction error (top) and mean depth-normalized triplets correct (bottom) reconstruction accuracy for simulated phylogenies with LMs drawn from either an indel distribution with n = 9,662 LMs (crosses) or a uniform distribution with eight LMs (dots). Trees were simulated varying key experimental parameters: the number of cells (far left), fraction of edit sites with an LM (middle left), the number of edit sites (middle right), and detection rate (far right) and reconstructed using UPGMA (blue), Neighbor joining (NJ; red), and Greedy (green) algorithms. The Greedy solver was less accurate in reconstructing trees with eight LMs due to increased homoplasy at individual edit sites, which rendered greedy splits suboptimal; in contrast, methods leveraging information from all edit sites– such as UPGMA and NJ–remained robust to homoplasy. Values held constant for simulations where the experimental parameter was not varied are marked with grey dashed lines. (**F**) Correlation of Robinson-Foulds reconstruction error and mean depth-normalized triplets correct accuracy for simulated phylogenies. Black line depicts regression with ribbon indicating 95% confidence interval. (**G**) Simulated LM accumulation at edit sites with varying edit rates (edits/day). (**H**) Identification of optimal edit rates for lineage tracing experiments of varying durations. Optimal edit site saturation lies between 60-80% (colored); in some cases, lower edit site saturation (50%) or higher (90%) may still allow for accurate reconstructions. (**I**) Mean fraction of branches in simulated phylogenies (n = 10) marked by any edit for different numbers of cells and edit sites given optimal editing kinetics. (**J**) *In silico* prediction of correct probe hybridization frequency as a function of LM insert length across simulated experiments with randomly sampled insert sequences. All simulations were run for ten iterations, with mean value reported; standard deviations shown as ribbons where appropriate.

A

*RNF2*


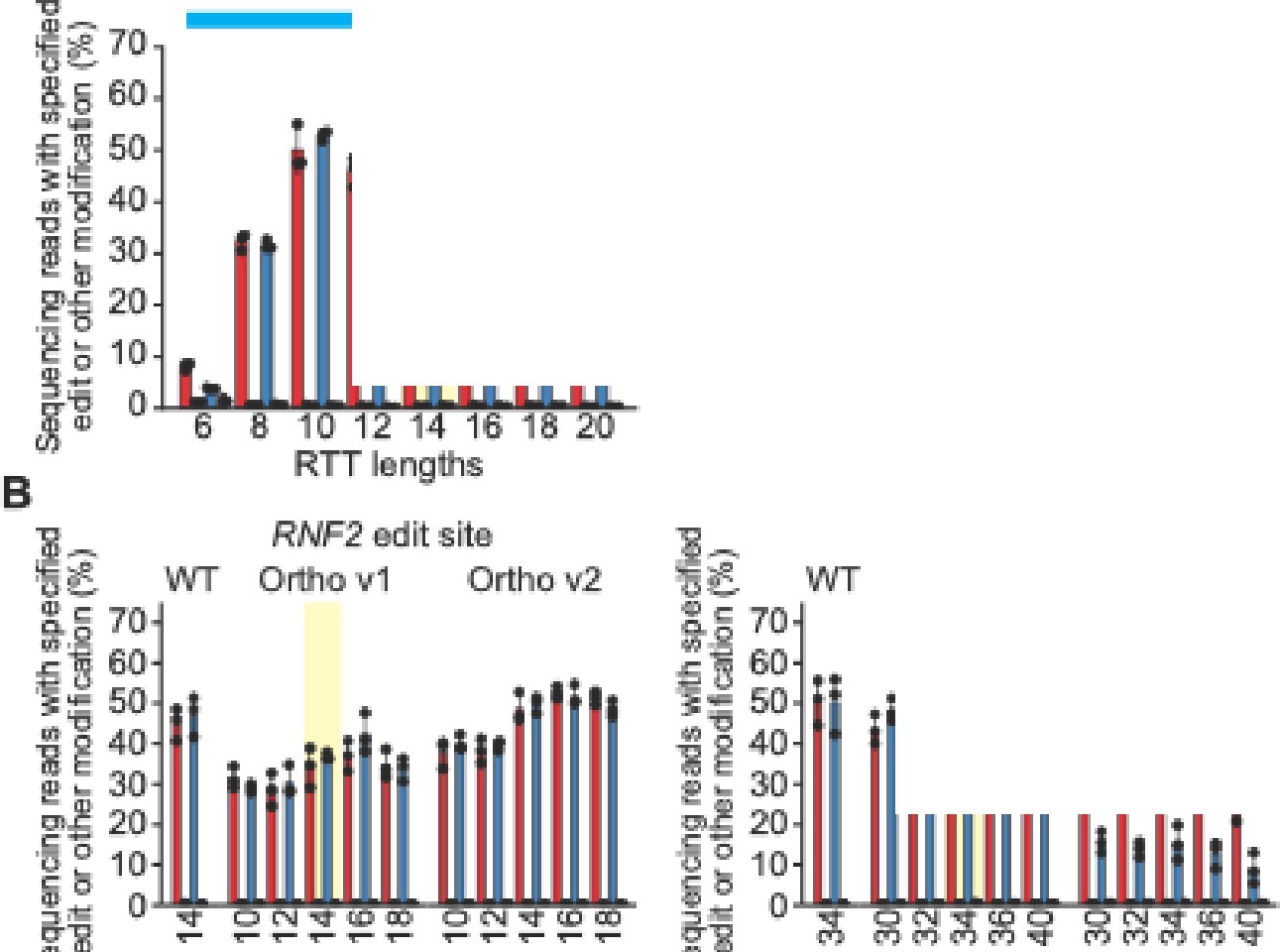

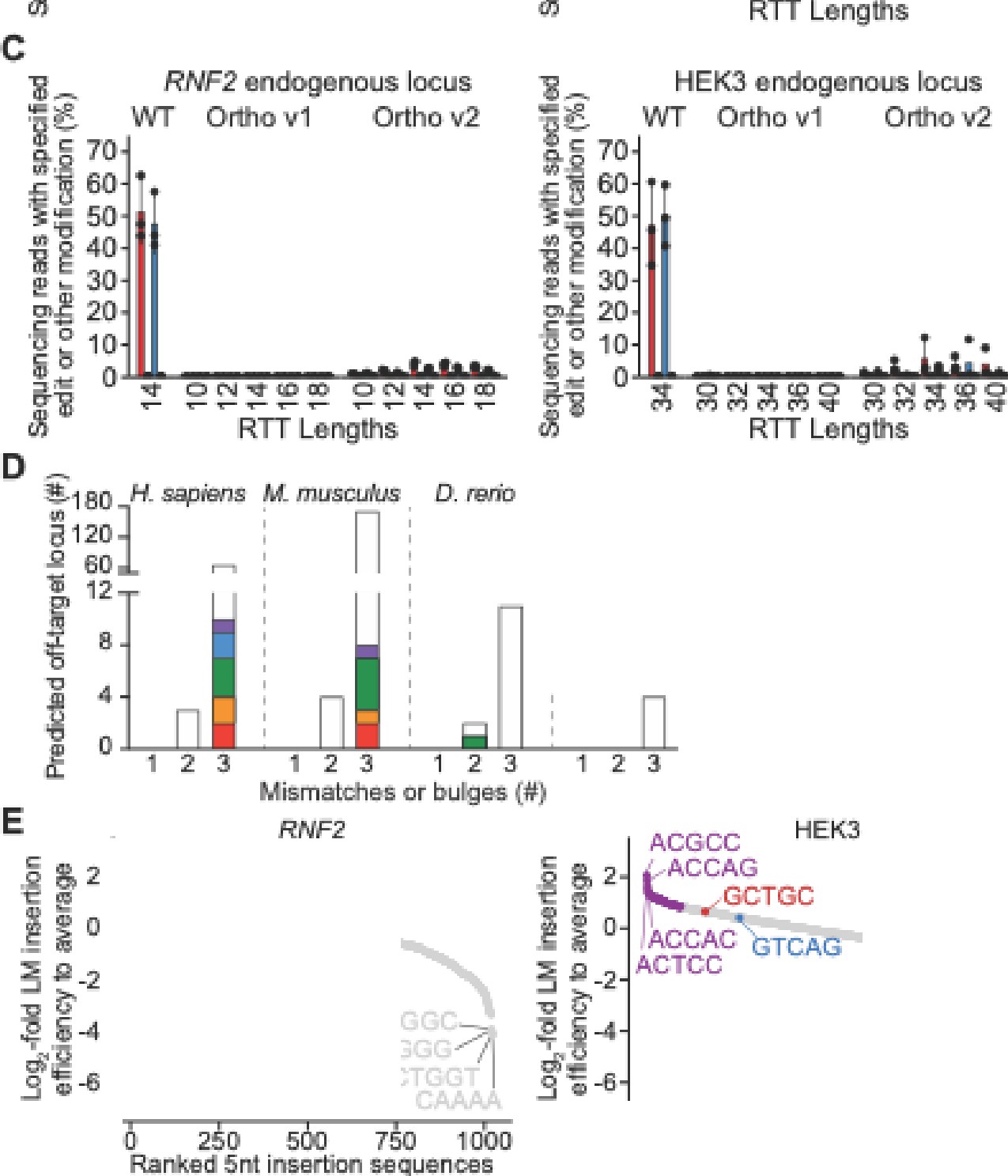

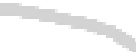


.:I

�ero

;§ a 5(1

fj'M

•

.

"

•

� .· 4(1

i l 3(1

�: 2(1

1! � 10

*i*^!^*-;* 0

J

lj *.i*

RTTlengh

**HEiK3** edit .site

'

Orlho **v1** Orlhov2

RTT Le,riglhs

n m�ogB'stl3.r

-Exon

Noo-cocfing

c::::J 5" UTR

-l?'ramolatr

-TTS

-3"UTR

c::::J lnfery;anr<ll inlronic

.... GGGCA

�cm GTCAG

/ TGCGA GCTGC

ACTTT

er

C

***AAA)*** /

o 2so soo *nm* moo

Ranked Sn! in:serficn se,qLJBn□e:s.

- +GCTGC insertion ■+GTCAG inSl}'ff:ion

HEiK3

other

*EMX1*


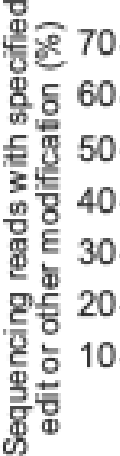


.

.

�

0

I

12�

1o7 a-------w, 22

�

RTTlenglhs


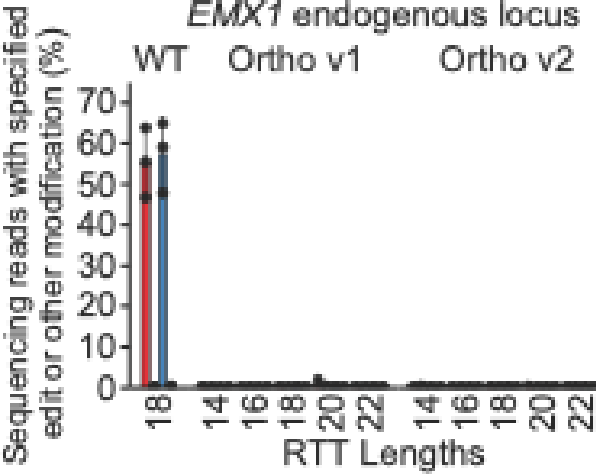


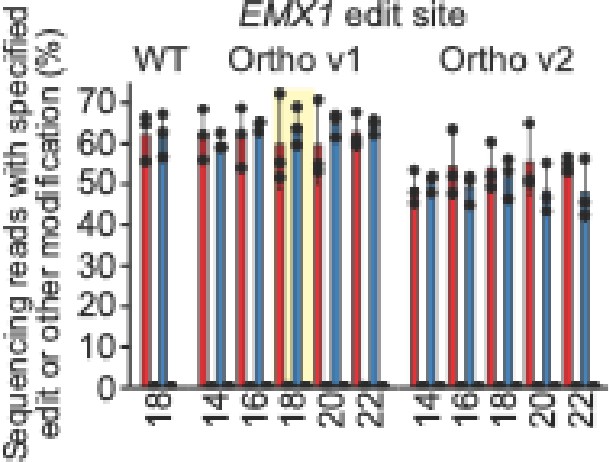


RTT Le,riglhs

*EMX1*


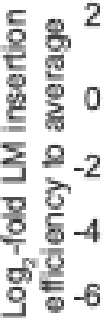

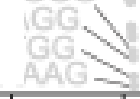


GGCAG

�TCG

j1ACTGG

ACCAT

GTCAG

GCTGC

*AN*

250 500 *7* •□, t

0

Ranked Sn! in:serfie>n se,qu:ence:s.

**Fig. S2. Edit site optimization and orthogonalization.** (**A**) Fine-grained optimization of reverse transcription template (RTT) lengths for epegRNAs installing two representative five nucleotide (5nt) lineage marks (LMs) at endogenous loci in HEK293T cells. Optimal RTT lengths for each edit site sequence highlighted in yellow. (**B**) Editing efficiencies for representative LMs at tracing cassettes comprising *RNF2*, HEK3, and *EMX1* edit site sequences from the human genome (WT) or orthogonalized in two ways (Ortho v1–seed sequence complemented, v2–seed sequence reverse complemented). Editing efficiencies for each edit site variant and its cognate epegRNA with varying RTT lengths are shown. (**C**) Editing of the endogenous *RNF2*, HEK3, and *EMX1* genomic loci in HEK293T cells when using WT, Ortho v1, or Ortho v2 epegRNAs. Mean of three biological replicates ± standard deviation depicted for (A-C). (**D**) *In silico* screening of potential genomic off-targets in *H. sapiens*, *M. musculus*, *D. rerio*, and *D. melanogaster* for Ortho v1 epegRNAs. Off-targets listed by species and sub-categorized by the number of mismatches or bulges in these off-targets. 18 putative off-targets in human and mouse non-intergenic regions were manually inspected for RTT homology and classified as unlikely to support prime editing. UTR = untranslated region. TTS = transcription termination sites. (**E**) Comprehensive LM screen comparing insertion efficiency of all possible 1,024 5nt insertions at three test loci in HEK293T cells. Log_2_-fold insertion efficiencies were calculated relative to input plasmid library and averaged across three biological replicates. Representative 5nt LMs used in (A-C) shown in red and blue, and 96 best-performing LMs highlighted in purple.

(")


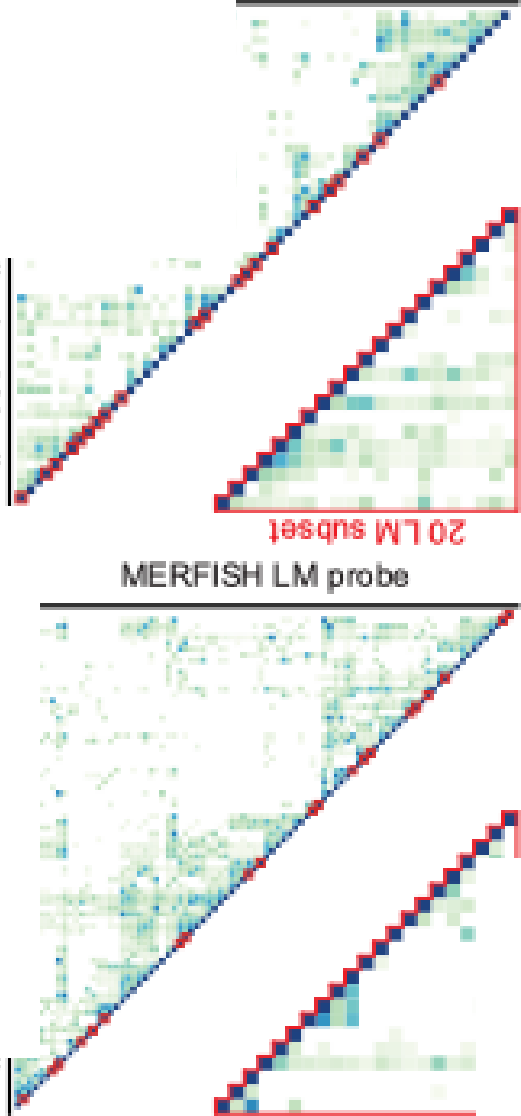


MER!Fl8H **LM probe**

�

..

;;;,,

�.

i

iii

[

[

·

**r­**

:§:

m

;;;,,

0.

�

;;;,,

!:!!.

iii

i

)"

[

�·

**r­**

:§:

di 1•.•

�

••


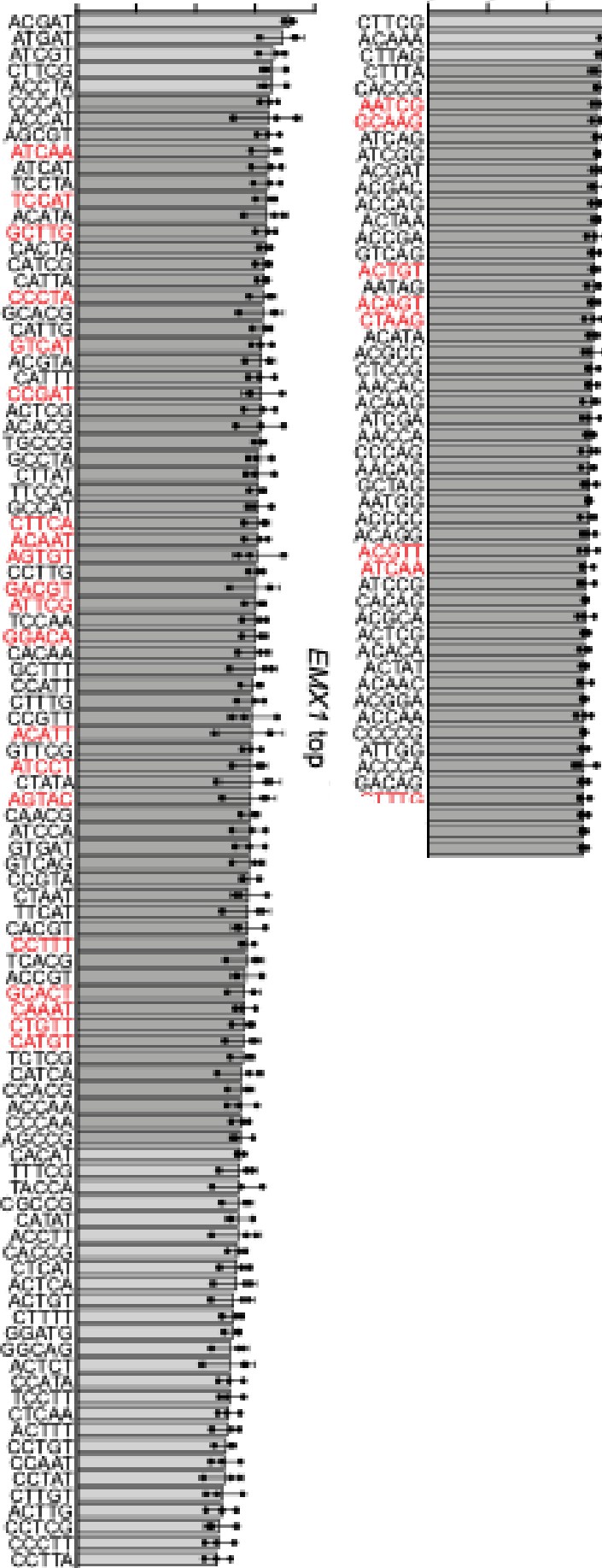

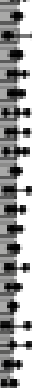


i.!, �At:TtCiT

.,, AeADC:

At:T.to.C

g NJf�

�

=AC:TTC

CCL',,l

t.&lli�

™

AC:C:C:T

�

13JJI.Jl

AC:C:AT

Arrr,,A

ATCTC

=c:

A.CllOO

=ACC:TT

l=lOOCC

=Arew:.

=

CTt:rc

=

ACCT.A

CCC:M

AA.CIT:

Al'TTA ACATC A\TOT!l

ACC:ll.a

A□c:rc

=CT

w;rrCA.t-----

�

**0**

�I

�

t,j

**0**

**0** �**0**

l>-

"'

00

**0**

.,.

l□CA

�

�CT.�G�

-::+

A,Y.

'

re

�

;cm;

�3CTI

��-

rec

;c3TTI;'.;T_..C

llT

--=

--

,.

iCTCC

��i_gl

,-

�CA(lffi

!:ll

::c

�

"Q

f

�

0

.:,i,'

��I

rt;e;;tc;t;CI

"'ClC[;

'ICCfTCCfCil

I�

�

.f,',

**m**

��

=T ..T. .�..

.

.�

�,

-

!"

*l*

!

**00**

TCff,\

1/11

'i"i;

**5,'**

;�

.::,:,

;;i.

;;;·

�1c

�T,c;_·.c.

.cG

;c{OO

CC

re

�

�

b� l'

,cr.oo

�

....

"nOeOCeC

ffc;,,,fi

Alli�

�ATTCi!I

T.

**'l"**

...

�

�**fl.lfl.l.l..i**

�

-

C�:

GTAACI

I

·I

.�'"-

,,.

'CCTC

�

**tu** Sequen;I19 read$��cjfied edit(%) ►

SoqUtJncing reads: **'nilti** specified edit or Olhcr mooificatoo {%J

""l\lWl>-Olt,:J-.JOOIJ:J

0000000000

ATTAT�• • �

ATGCT

GGAGT

e, � � � �

e, � � �

AAGGC

A'f,i!,18 '�

..

AU.Ml"

TAAGT

*E:C,.*

•

COOCA ACTGT CTGAC

tt�

GAA.GC

J�1

_#,TAT

mm;11

TGAT/t

�

.::,;,,

i'

�

�

.::,;,

i'

&quermg read&mili spalied

o11ie"

edit or mooiooalirn ('¾)

_,""..,

I

.,,.,,'-Ja,oc

'F'ooe,e,e,e,??�

ACAGl"

Cl M:CGT

�. i�gi

...

ID CGGGl"

c. CTTGC

·•

[ CGATT

::;, cl�

....

c;;c;;cc;;cj

.

0

!ll

!:f

�II

�811

CCTAT]I

G.AAJ\Gl

GOTGG¢TAGTII

fu���I

.....

•

..

m

�

l!l,

""

im

,

[

�

.::,;,

i'

**t»,:,)**


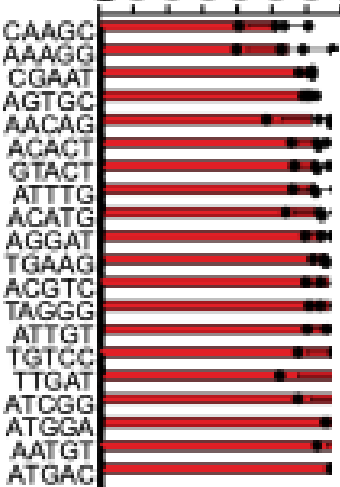
Soquonmg reads Yoiti'I specified ooit or *o*l'Ftn"modlfication {%*J* 00 r:,:�� gi 1!l 2l�1g

�

;;;,,

- - **O'I·**

iji'

�

�

.::,;,

i'

# i

ff

Qf;I

r­

:§:

i

;;;,,

ia1.qn,s l/111 0�

MER!FISH **LM** probe


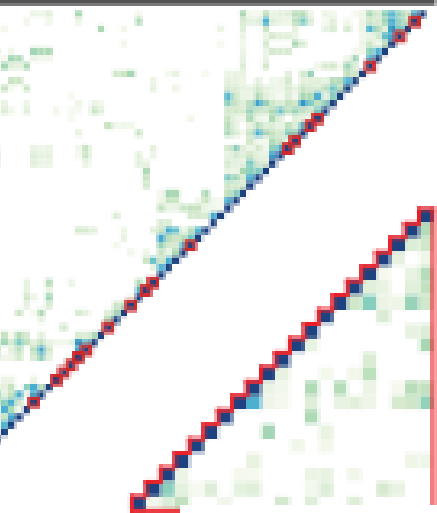


iesqn.s li'll1 o�

� **N** � � � �

(J<>OIJIY.<ll. =�';>Ve,

�

.::,;,

i'

*w*

7

**Fig. S3. Screening for balanced and orthogonal LMs.** (**A**) Installation efficiencies for best- performing five nucleotide (5nt) lineage marks (LMs) for each edit site. Mean of three biological replicates ± standard deviation depicted. *RNF2* = 85 LMs, HEK3 = 94 LMs, *EMX1* = 96 LMs. Complete set shown, those within 10% efficiency used for detection specificity measurements in

(B) highlighted with dark grey bars and final 20 candidate 5nt LMs retested at orthogonal edit sites in (C) highlighted with red text. **(B)** Predicted probe hybridization specificity measurements (Gibbs free energy = ∆G) for discriminating all best-performing 5nt LMs from one another (bottom) or selected 20 LM subset with reduced cross-hybridization (top). (**C**) Editing efficiency of 20 LM subset for each edit site with optimized cross-hybridization retested at orthogonal edit sites in HEK293T cells. Mean of four biological replicates ± standard deviation depicted.


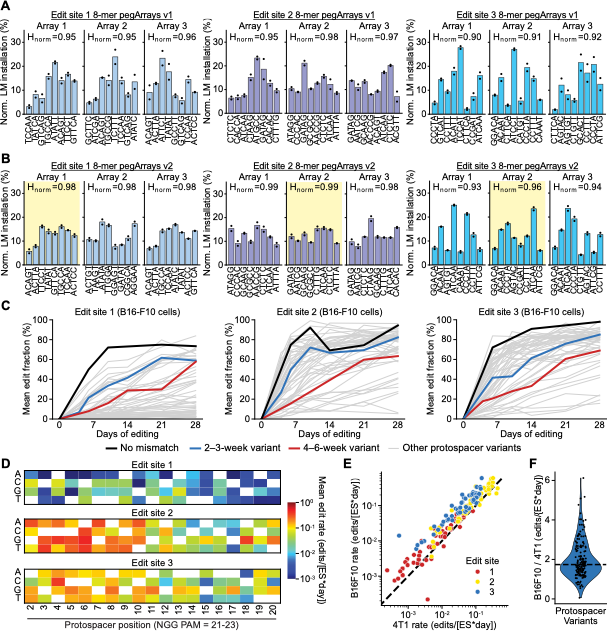


**Fig. S4. pegArray optimization and kinetic tuning.** (**A**) Normalized (Norm.) lineage mark (LM) installation efficiencies for the first iteration (v1) of 8-mer pegArrays for edit sites 1, 2, and 3 in B16-F10 cells. Three arrays were tested per edit site. H_norm_ values provided for each 8-mer. Five nucleotide (5nt) LM sequence listed under its position in the 8-mer. (**B**) Normalized LM installation efficiency for the second iteration (v2) of 8-mer pegArrays for edit sites 1, 2, and 3 in B16-F10 cells averaged across two biological replicates. The final 8-mer used in the concatenated 24-mer is highlighted in yellow. Two biological replicates for (A) and (B). (**C**) Editing kinetics for protospacer mismatch variants at edit sites 1, 2, and 3 in B16-F10 cells. Labeling as in (Fig. 1G); grey lines show all protospacer variants tested; no mismatch protospacers shown in black; protospacer mismatches selected for 2–3-week timescales shown in blue; 4–6-week protospacer mismatches in red. (**D**) Mean estimated edit rate associated with indicated protospacer mismatches along the length of the protospacer for each edit site. Protospacer mismatches decrease editing rates across three orders of magnitude in two tested MMR-competent cell lines (B16-F10 and 4T1) with slower editing rates for mismatches closer to the protospacer adjacent motif (PAM). The original base at each position is a white box in the heatmap. (**E**) Editing rate comparison for each variant in 4T1 and B16-F10 cells. Points colored by edit site. Dashed line depicts y = x as a guide.

(**F**) Edit rate increase in B16-F10 cells relative to 4T1 cells for each protospacer variant with dashed line indicating median increase.


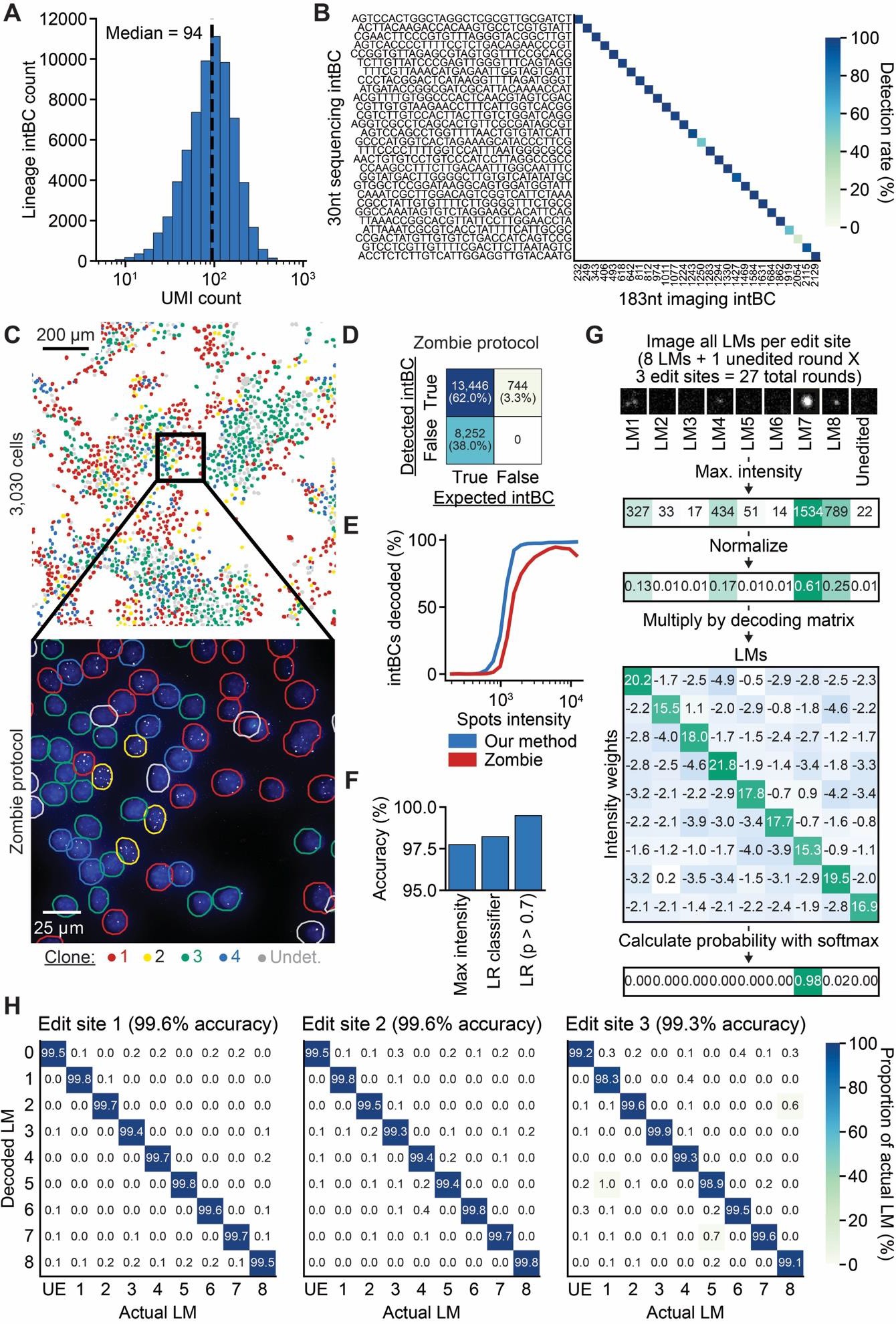


**Fig. S5. Optimization of *in vitro* PEtracer validation experiments.** (**A**) Histogram of unique molecular identifiers (UMIs) per lineage cassette integration barcode (intBC) detected in fully- edited cell clones by droplet-based single-cell RNA-seq (scRNA-seq). (**B**) Mapping of 30nt sequencing intBCs to 183nt imaging-decoded intBCs by scRNA-seq. (**C**) Wide field of view (top) and higher magnification view (bottom) for experiments using T7 *in situ* transcription and imaging following the Askary *et al.* published Zombie protocol (*56*). Cells are colored by their clone identities where clone 1 = red, clone 2 = yellow, clone 3 = green, and clone 4 = blue with undetermined cells (Undet.) that could not be confidently assigned to a clone shown in grey. DAPI nuclear staining (blue) and the two common bits (C1 = green; C2 = magenta). Integration amplicons appear as colocalized common-bit puncta (white). (**D**) Confusion matrix for intBC decoding using the previously-published Zombie *in situ* T7 protocol. (**E**) Decoding efficiency as a function of spot intensity for *in situ* T7 polymerase-generated spots representing integrated lineage cassettes using our new protocol (blue line) versus Zombie protocol (red line). (**F**) Imaging-based readout accuracy of lineage marks (LMs) using the maximum (Max.) spot intensity, the logistic regression (LR) classifier, or the LR classifier with a p >0.7 cutoff with 5- fold cross validation. (**G**) Logistic regression classifier decoding workflow from images to LM assignment probabilities for an example spot and edit site. (**H**) Confusion matrix depicting the decoding accuracy with 5-fold cross validation for each individual LM and the unedited (UE) state using the PEtracer *in situ* T7 amplification protocol. For each intBC in each cell, the “actual” LM from the ground-truth intBC-LM mapping is compared to the decoded LM from the processing workflow in (G).


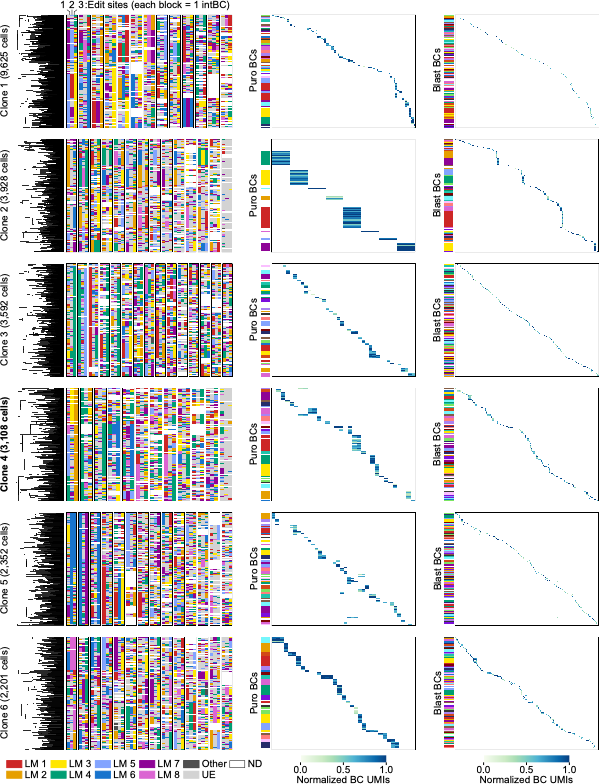


**Fig. S6. Phylogenetic reconstructions with paired puromycin and blasticidin barcode data.** Character matrices and phylogenies for the 6 clones assayed in the single cell RNA sequencing *in vitro* barcoding experiment reconstructed with the neighbor joining algorithm and PEtracer evolving lineage marks (LMs) along with heatmaps of normalized unique molecular identifier (UMI) counts for Puromycin (Puro) and Blasticidin (Blast) static barcodes (BCs). Clone 4 is bolded as it is highlighted in the main text. Imperfect static BC groups indicated as gaps in called groupings. Notably, imperfect calling of barcode groups was partially responsible for FMI scores <1. Integration barcodes are denoted as black rectangles comprising three associated edit sites colored by LM identity. Color key for LM identities provided in bottom left; UE = unedited, ND = not detected. Puro and Blast BC groups based on normalized BC UMI counts denoted as colored blocks to the Puro BC and Blast BC heatmaps.


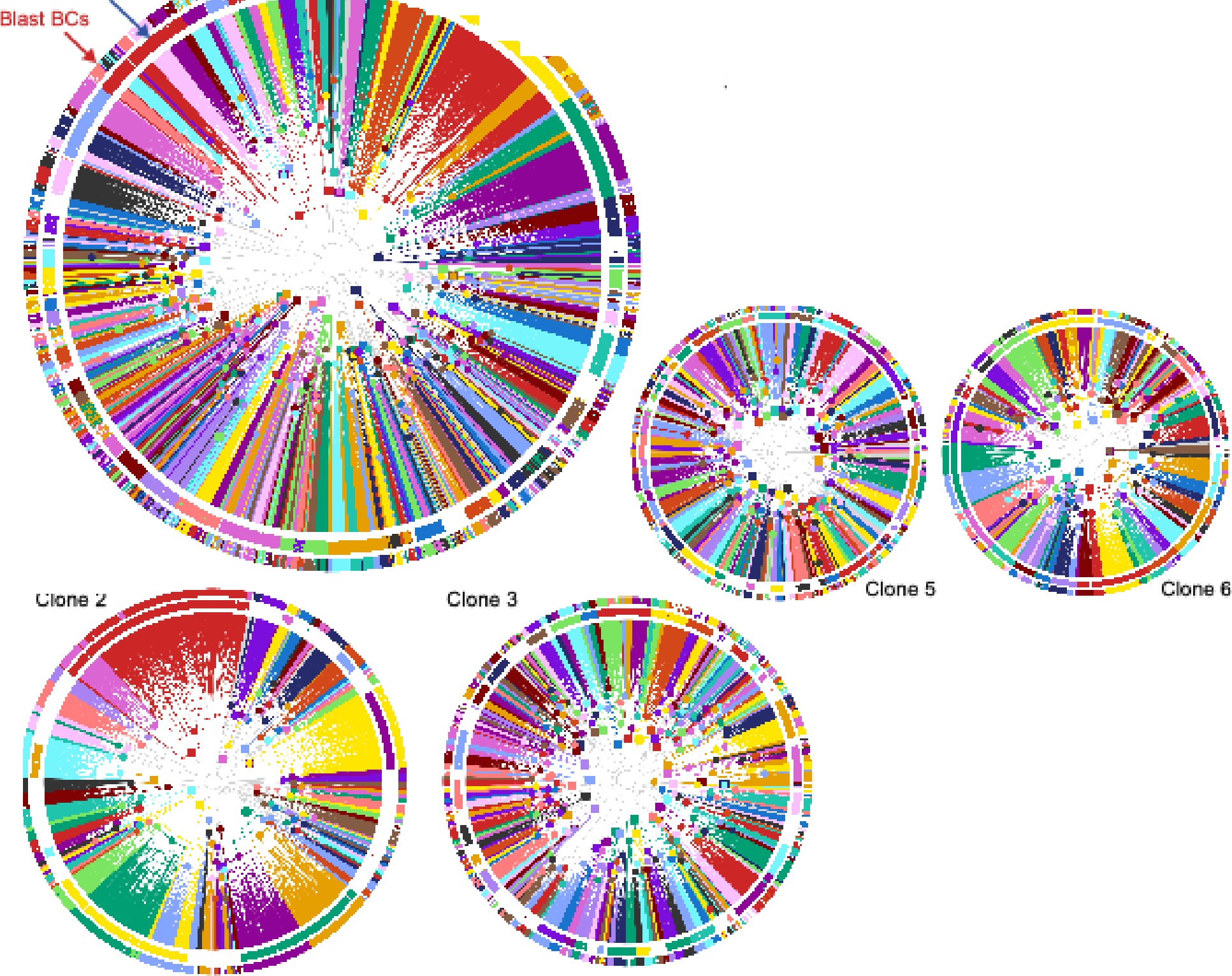

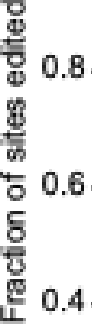

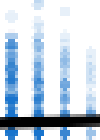


**A**

Inferred LCA

Pu

'\�,...

'

- Blast BC

,,.,

a.

•■ PuroBC

o?i U·

·QI

Qj•

j o?

qi,-

cf �

0

Ill

,,

(.)

i

-S*

"C•--

�

u

Jc

-.:,:Iii :=:j

�i

liD ..,j'e l"l:ll

�Ill <3!

w;;:

gi QI

�

I:

**.ii**

:er· ^li^ ^�^

;:i,

JIil;

_,.; <:!:'.'Cl **Q. !W**

g,....,.. a;i..,.,,

9,625 *n.n* B6.06I11.32 0.92 O.Bi!i 5.86 7.99•

2 3,928 62.64 B7.39 8.76 1I.OO 0.96

3 3,1592 7i5.2A B8.16 10.03 0.96 0.94

4 3,rn8 72.117 B7.77 9.12 0.96 0.92

2.95 7.29•

5.07 8.38

4.88 7.4'6

5 2,352 72.01 B7.64 10.03 0.90 0.90 5.48 7.6B

6 2,201 68.66 B9.91 8.511 0.95 0.91 5.08 8.01

Clone 1

B

11

••

rt=O.OD

I

0

I

5

I

10

PEm:aix expre,ssioo


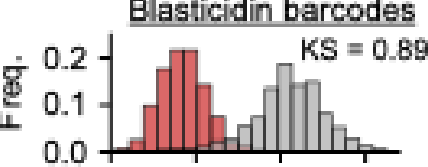


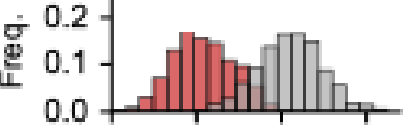


C

IPUn:mlY.Cjtl ba:rcpd&s;

KS• 0.80

8


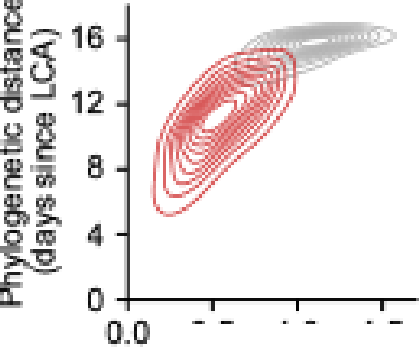

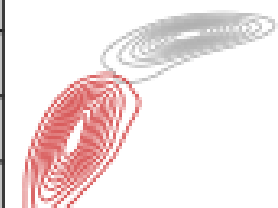

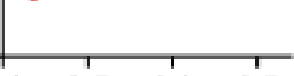

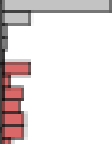


KS •0.96

1

<'16

(.)

-g-' 12

ii

8

t� 4 KS• O.l!l8,

p,

a. 0

D � �

- 1. ' .0 ' .5 0.0 0.5


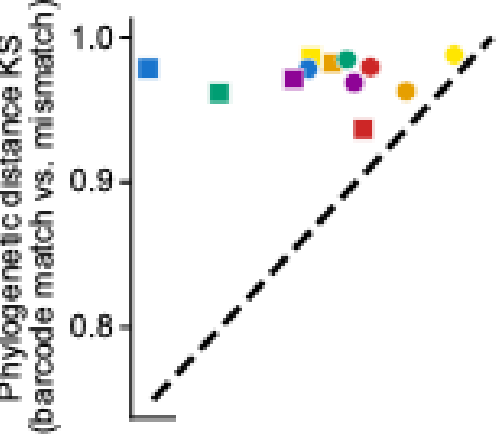


-1 3-5

2-4-6

■I Puro

e

Blasl

LM diis.tanoe Freq. (Hammi g)

- - - IBarcode matcll

0.0 D.5 .0 ' .5

LM ditS.tanoe (Hemmirng)

- IBarcDde ,ismalch

0.0 0.5

Freq.

D.8 O.Q 1.0

LM cfistar\ce KS

{barwde matcli v.s. mismatcl1)

**Fig. S7. Characterization of phylogenetic reconstruction performance with scRNA-seq PEtracer data.** (**A**) Reconstructed phylogenies for clones 1, 2, 3, 5, and 6. Coloring and plotting identical to Fig. 3D where rings are colored to show puromycin (Puro; inner ring) and blasticidin (Blast; outer ring) static barcode (BC) groups for each cell (white indicates missing data). Phylogeny branches colored to match BC assignments. The inferred lowest common ancestor (LCA) is marked and colored to match each BC group (Puro = square; Blast = circle). Embedded table (top right) shows useful statistics for each phylogeny. Average = avg. (**B**) PEmax expression versus fraction of sites edited for clone 4. Black line depicts regression with ribbon indicating 95% confidence interval. (**C**) Pairwise LM distance versus phylogenetic distance for cells sharing (red; barcode match) or not sharing (barcode mismatch; grey) puromycin (left) and blasticidin (right) static barcodes in Clone 4 (main text phylogeny). Kolmogorov-Smirnov (KS) statistic for the separation of matched and unmatched barcode distributions reported for each BC and distance metric. Freq. = Frequency. (**D**) Pairwise LM versus phylogenetic distance KS statistics for puromycin (square) and blasticidin (circle) BC groups across all 6 clones tested in this experiment. Dashed line depicts y = x as a guide.


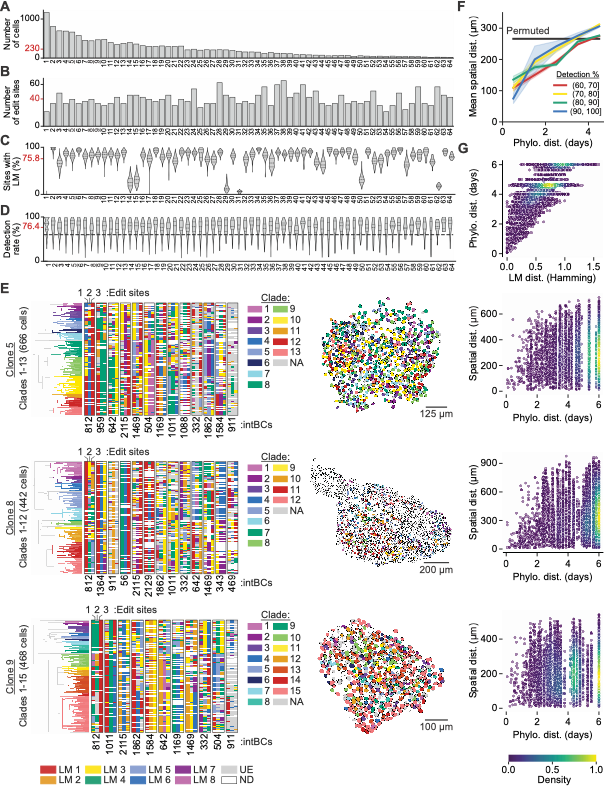


**Fig. S8. Evaluation of phylogenetic reconstruction performance with imaging-based PEtracer readout.** The number of cells (**A**), number of edit sites (**B**), % of sites with a lineage mark (LM) modification (**C**), and the integration barcode (intBC) detection rate (**D**) for 64 evolving clones of 4T1 cells imaged on a single cover slip. Average value for each plot marked in red on the y-axis. Dashed lines on violins indicate median and dotted lines indicated 25^th^ and 75^th^ percentiles. Clone 3 is bolded as it is shown in the main text, Clones 5, 8, and 9 are underlined as they are depicted throughout this figure. Cells with detection rates <60% (indicated with dashed line in D) were excluded from downstream analyses. (**E**) Data for clones 5, 8, and 9. Left shows the character matrix and phylogeny for each clone. intBCs are listed below each character matrix where the three edit sites are colored by LM identity; UE = unedited, ND = not detected. The number of cells in each clone is denoted and the phylogeny is colored by clade. Clades are listed for each clone and are used to pseudo-color nuclear masks for the colony shown in the middle. NA

= not assigned. Grey cells = not assigned a clade; White cells = not included in phylogeny. Pairwise phylogenetic versus spatial distance for each clone provided on the right. Phylo. = phylogenetic and dist. = distance. (**F**) For clone 3, mean pairwise phylogenetic versus spatial distance for cells grouped by detection rate decile. Ribbons show standard error and black line is the mean distance between randomly permuted pairs. (**G**) For clone 3, pairwise phylogenetic versus LM distance.


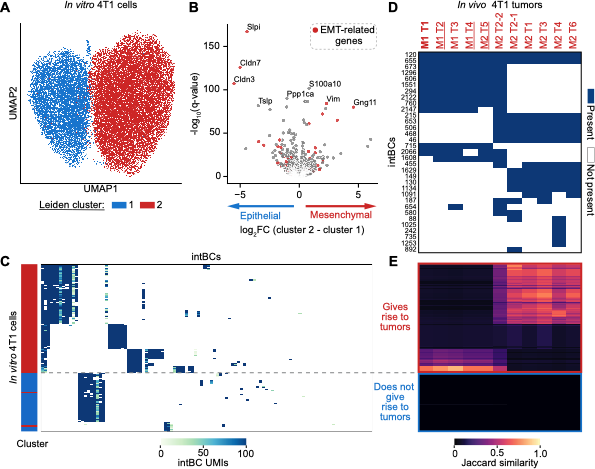


**Fig. S9. Linking pre-injection *in vitro* cells to *in vivo* tumors**. (A) UMAP embedding colored by Leiden clusters for 21,644 sampled cells from *in vitro* 4T1 population used to seed tumors. (B) Differentially expressed genes between Leiden clusters with EMT-related genes highlighted in red. Statistical significance (q-value) determined with Student’s t-test and adjusted for multiple comparisons with the Benjamini-Hochberg procedure. (C) Unique molecular identifier (UMI) counts across detected intBCs for the three dominant *in vitro* 4T1 cell clones (with numerous sub- clonal populations derived from successive rounds of engineering which introduced population bottlenecks). Color bar indicates Leiden cluster assignment of each cell. (D) Detected intBCs for *in vivo* tumors from mouse 1 (M1) and mouse 2 (M2). Blue indicates an intBC is present in the seeding cell, white indicates that intBC was not detected. Tumors included in lineage analysis (M1 T1, M1 T2, M1 T4, and M2 T5) share a set of common intBCs, suggesting they derive from a common clone. Unique combinations of intBCs may reflect sub-clonal populations or imperfect intBC assignment *in vivo*. (E) Jaccard similarity between set of detected intBCs for each *in vitro* cell and *in vivo* tumor pair, highlighting a dominant *in vitro* clone that gives rise to tumors *in vivo* as well as two clones that do not give rise to tumors.


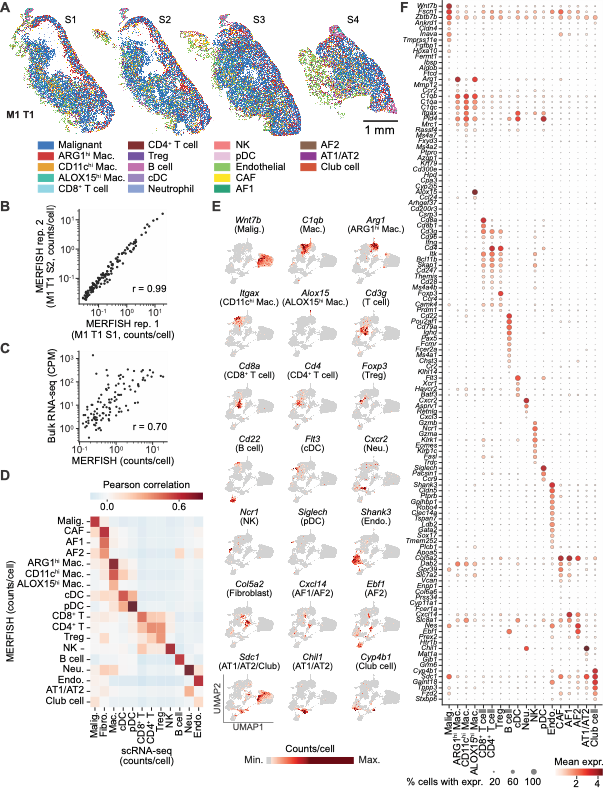


**Fig. S10. Reproducibility and additional markers for MERFISH transcriptomic data.** (**A**) Spatial organization of cell types across four sections (S1-S4) of Mouse 1 Tumor 1 (M1 T1). Cell types colored to match Fig. 4. Scale bar = 1 mm. (**B**) Replicate correlation of transcript counts per cell of individual genes detected in MERFISH data acquired from M1 T1 S1 and M1 T1 S2. (**C**) Correlation between MERFISH counts per cell of individual genes across M1 T1 and bulk RNA- seq data for 4T1 lung metastases from Ferrer *et al* (*106*); r = Pearson correlation. (**D**) Pairwise Pearson correlation of mean counts per cell from our M1 T1 MERFISH dataset with mean counts per cell in scRNA-seq data generated from 4T1 primary tumors as well as lung and liver of tumor- bearing mice. (**E**) UMAP embedding of all 368,722 cells from Mouse 1 and Mouse 2 tumors colored by marker gene expression level for each of the 18 distinct cell types annotated with this library. (**F**) Gene expression (expr.) profiles for all 124 probed genes across the 18 cell types resolved with this library. Dot color indicates the mean gene expression for a gene within a cell type while dot size indicates the percent of cells within a cell type that express that gene. Cell type abbreviations are as follows: Malig. = malignant, Mac. = macrophage, Treg = regulatory T cell, NK = Natural Killer cell, cDC = Conventional Dendritic Cell, pDC = plasmacytoid Dendritic Cell, Neu. = Neutrophil, Endo. = endothelial, CAF = Cancer Associated Fibroblast, AF1/AF2 = Alveolar Fibroblast Type 1/2, AT1/AT2 = alveolar epithelial type 1/2.


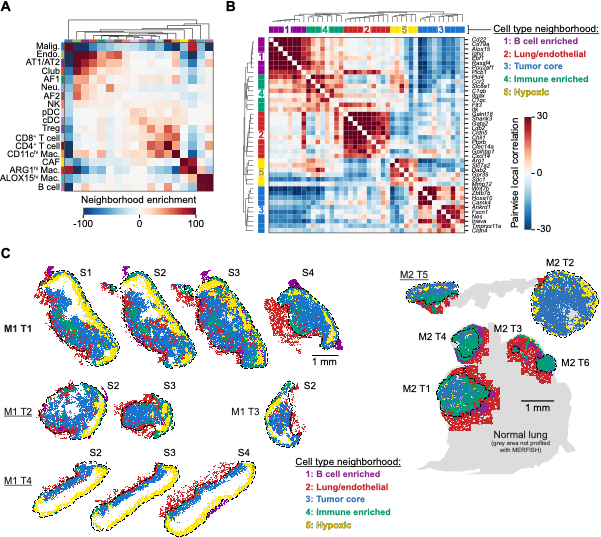


**Fig. S11. Spatial neighborhood analysis of cell types in 4T1 tumors.** (**A**) Cellular neighborhood enrichment (k = 6 closest neighbors) for cells in sampled tissues across M1 and M2. (**B**) Pairwise local correlation of gene expression MERFISH counts between cell type neighborhoods identified by Hotspot for tumor M1 T1. (**C**) Spatial distribution of cell type neighborhoods identified by Hotspot for all tumors and all sections.


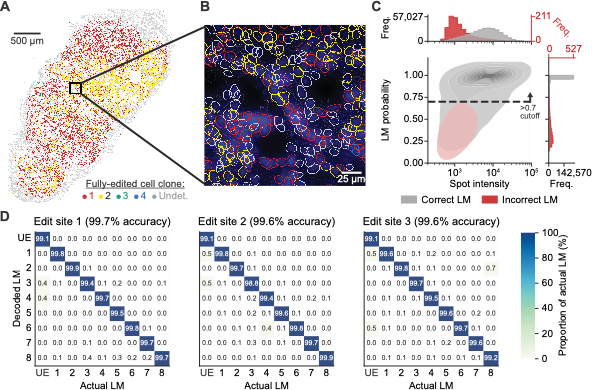


**Fig. S12. *In vivo* lineage mark detection using fully-edited 4T1 cells.** (**A**) Spatial organization of fully-edited 4T1 cells seeded in the mammary fat pad of a female mouse. Nuclei are colored by their clone assignments, matching Fig. 2. Clone 1 = red, clone 2 = yellow, clone 3 = green, and clone 4 = blue and grey = undetermined (Undet.) and non-malignant cells which do not contain the PEtracer components. Scale bar = 500 µm. (**B**) Higher magnification view of a field of view in this tumor with DAPI nuclear staining (blue) and nuclei outlines colored by clone assignment. Scale bar = 25 µm. (**C**) Validation and performance of the logistic regression classifier for LM assignment from *in vivo* imaging-based PEtracer data. Assignment probability cutoff of p >0.7 for decoded LMs is noted as a dashed line. LM = lineage mark. Freq. = frequency. (**D**) Confusion matrix depicting the decoding accuracy for each individual LM and unedited (UE) state *in vivo*. For each intBC in each cell, the “actual” LM from the ground-truth intBC-LM mapping is compared to the decoded LM.


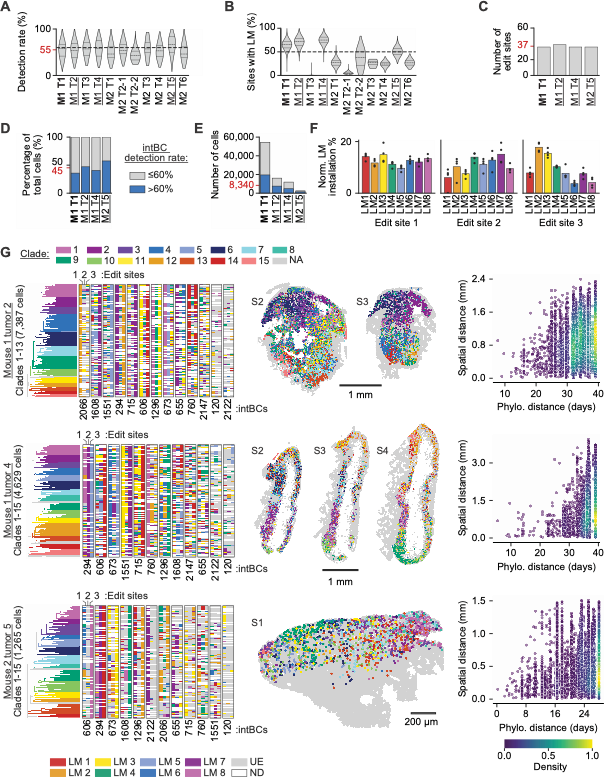


**Fig. S13. *In vivo* phylogenetic reconstructions with high-resolution MERFISH-based imaging data.** The detection rate (**A**) and fraction of sites with lineage mark (LM) modification

(**B**) for all tumors across Mouse 1 and Mouse 2. Tumor clones with mean editing fractions <50% (indicated with dashed line in B) were excluded from downstream analyses. Dashed lines on violins indicate median and dotted lines indicated 25^th^ and 75^th^ percentiles. For four analyzed tumors, the number of edit sites (**C**), the percent of total malignant cells (**D**), and count of malignant cells (**E**) that satisfy our filtering criteria and are therefore included in phylogenies. Average value for each plot marked in red on the y-axis for (A) and (C-E). The M1 T1 phylogeny is shown in Fig. 5**,** while phylogenies for M1 T2, M1 T4, and M2 T5 are detailed below in (G). (**F**) Normalized LM installation efficiency for all eight LMs at each of the three edit sites based on LM transition probability along branches. Mean of four analyzed tumors depicted. (**G**) Left shows the character matrix and phylogeny for the cells with >60% integration barcode (intBC) detection for each tumor. intBCs are listed below each character matrix where each edit site is colored by LM identity; UE = unedited, ND = not detected. LM color assignment is listed at the bottom and is consistent with (F). The number of cells in each clone is denoted and the phylogeny is colored by clade. Clades are listed and colored at the top left. NA = not assigned. All cells in the phylogeny across multiple sections (S#) are pseudo-colored by their clade assignment in the middle. Some tumors were not in all sections. Seeding of individual tumors by a single cell is supported by the presence of different clonal edits, for example intBC 673 is associated with clonal edits ES1 LM3 (light purple) and ES2 LM5 (light blue) in mouse 1 tumor 2, ES1 LM6 (dark blue) and ES3 LM5 (light blue) in mouse 1 tumor 4, and ES3 LM3 (yellow) in mouse 2 tumor 5. Pairwise phylogenetic (Phylo.) versus spatial distance for each phylogeny is provided on the right. Scale bars provided for each tumor.

**B**


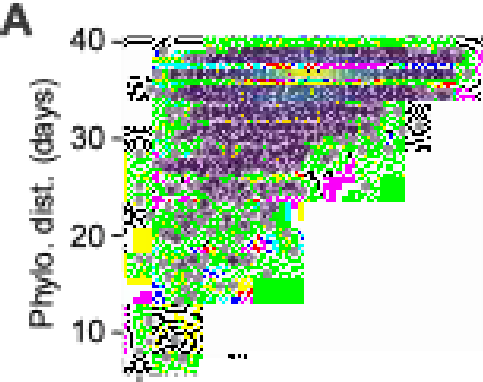


-,�--,,-.:i {■f

o_o 1.0 Ui

!3.0

i

j H5

*,*a*:n*

**C** Local LM diva�Ely (mean Hamrillmg)


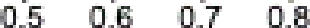


ll-4

F'.ainYi:seLM clisl. {Hammin!J)


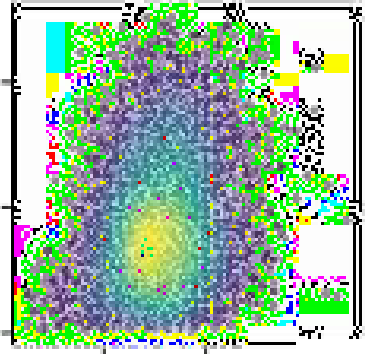


*v·* .

0.0

(HJ

**IHi**

Dansil'i'

0_5 Ul Ui


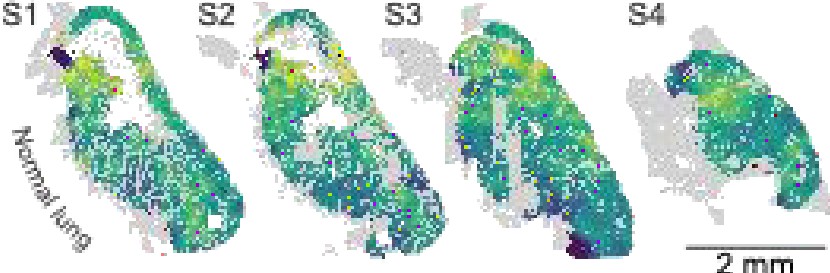
F'airwise LM d'isl. �mming) 1.0

**D** Line.age marks (lMs)

-UM 1- LM3-UM 5-LJM7 UE

U. 2-LJM4-l!M6-LJM5DNO

Cliidalsu�cla.de:

123:E<HI Siles Clade, 17-18 zaom-iri (1,187 cells)


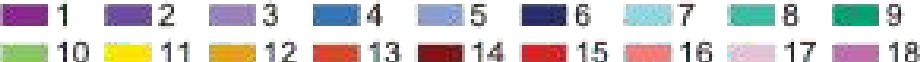


NA


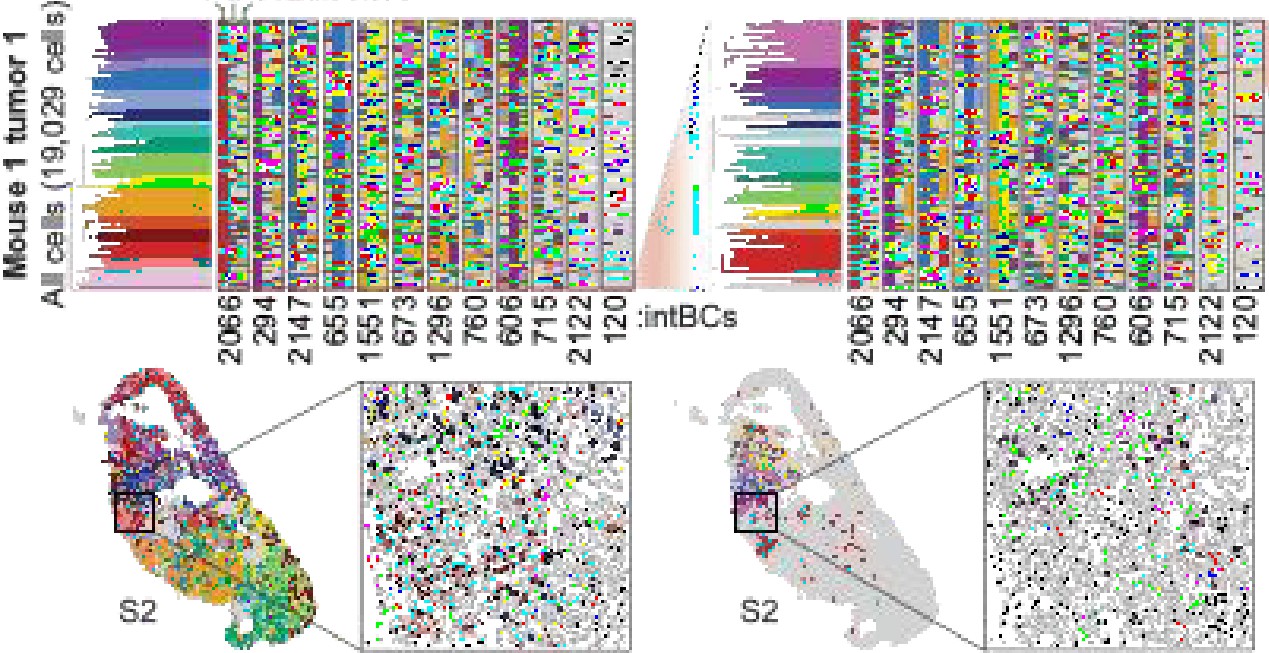


E

,,,**r..iiii**S**il**u**l**b**i**-**i**cl.ada 1 z.ocm-in (210 cells)

,^�^,


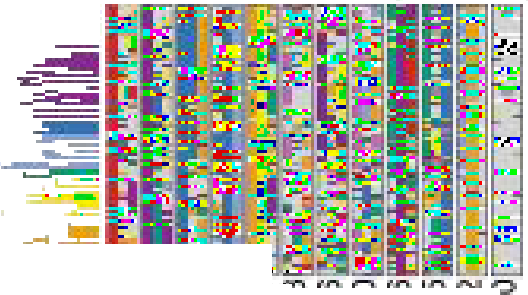


- ' '

---t�1' ..

\�

"�'.-

s*&1:* t2 �!a�ie� r::��

� N **N**


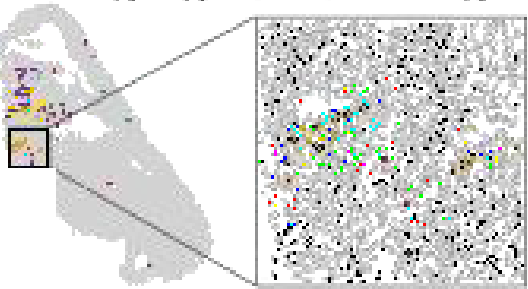


52

5001c1m 100 µrn

F **G**


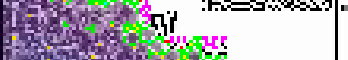


6

_sij


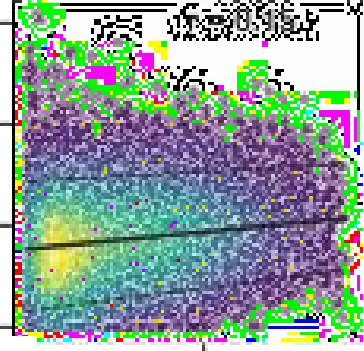

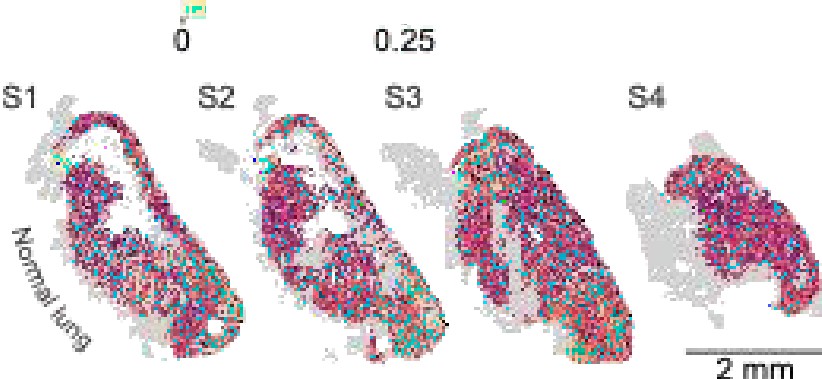


0.5

,�

**0.6**

5001Jm

1001,1m

**H**

5001-1m 1001,1m

Mean nei!Jhboor LM dislance

H 4,

if

2


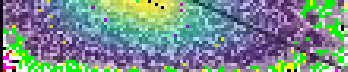


i�-�" **0.4**

�

lii 0.2

***.J***

0.0

K cOCdensity


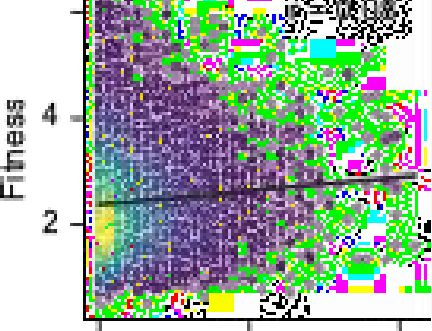


ll


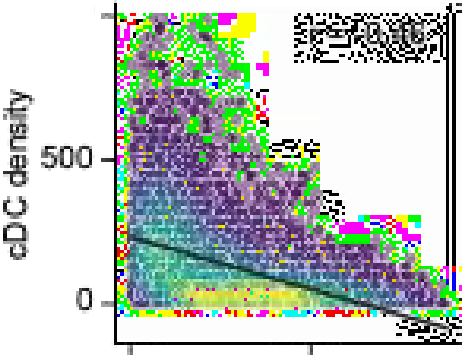


o_o

o,_5

| 0.0 0.2 | o_o | 0.5 |
| --- | --- | --- |
| Mean rieighbllif |  | Dist to rumor |
| LM dis.I. (t = 20) |  | boundary (mm) |
|  | 1000 |  |

*r-*

0 l75 750

0 500 1000

cDC delilSity

0.0 0.5

l:Jeh6.ity

Diel. ID IWlllllif

, oaunclEliJ'f {mm) 1_o


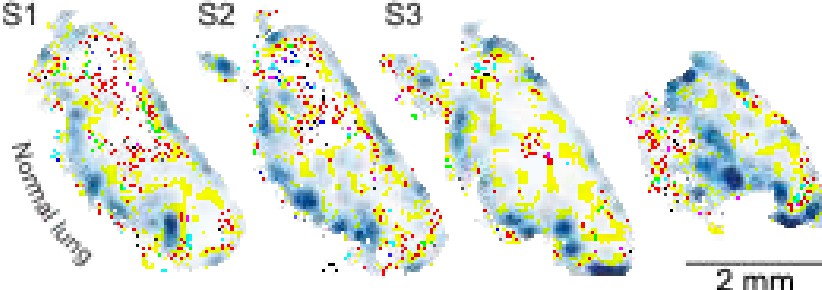


SIi

**Fig. S14. Phylodynamic analyses of tumor evolution *in vivo*.** (**A**) Pairwise LM versus phylogenetic (Phylo.) distance (dist.) for cells with >60% integration barcode (intBC) detection rate in Mouse 1 Tumor 1 (M1 T1). (**B**) Pairwise LM versus spatial distance for cells in (A). (**C**) Spatial organization of the local LM diversity, defined as mean pairwise Hamming distance within a 100 µm radius circle for all 19,029 cells in the M1 T1 phylogeny across four sections (S1 to S4)

(**D**) Character matrix and phylogeny zoom-in for cells in the M1 T1 phylogeny across four sections (S1 to S4). intBCs are listed below the character matrix, which is colored by LM identity for each edit site; UE = unedited, ND = not detected. Clades 1 through 18 are colored on the phylogeny on the left with a key at top right; NA = not assigned. Zoom-ins of smaller portions of the tree proceed from left to right. These zoom-in phylogenies are recolored by sub-clade to aid in visual inspection of the spatial distribution of assigned sub-clades for each sub-tree. (**E**) Spatial positions in section S2 of cells in (D) colored by their clade or sub-clade assignment (same color scheme as (D)). Scale bar = 500 µm for full section; scale bar for inset = 100 µm. Higher magnification inset shows nuclear segmentation masks. Grey circles denote malignant cells not assigned to a clade or not included in the sub-trees; white cells denote non-malignant cells. (**F**) Mean LM distance (Hamming) to 20 nearest neighbors in the character matrix versus fitness. Black line depicts regression with ribbon indicating 95% confidence interval for (F, G, I, J). (**G**) Dist. to the tumor boundary versus mean neighbor LM distance. Dashed lines indicate top and bottom decile. (**H**) Spatial organization of mean LM distance for all cells in the M1 T1 phylogeny across four sections (S1 to S4). (**I**) Conventional dendritic cell (cDC) density versus malignant cell fitness. (**J**) Distance (dist.) to the tumor boundary versus DC density. (**K**) Spatial positions of cells colored by local cDC density across M1 T1 tumor four sections (S1 to S4).


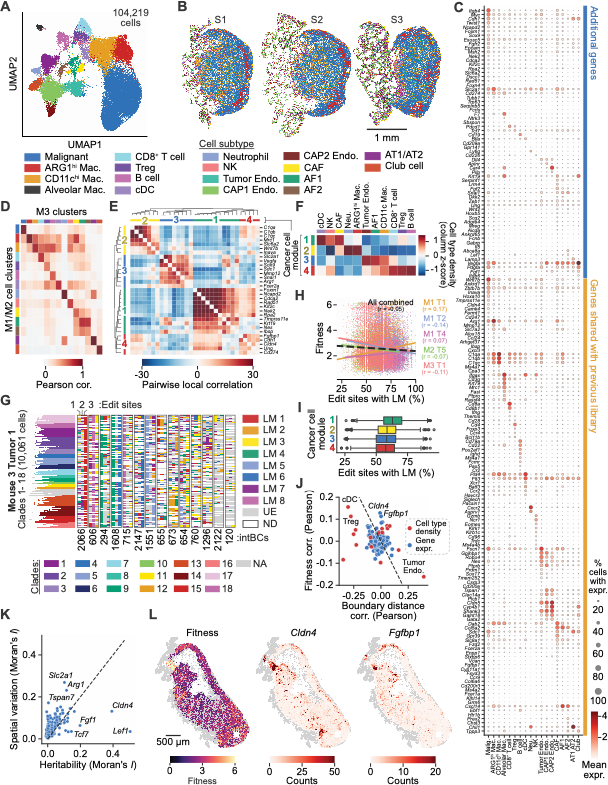


**Fig. S15. Consistent features driving metastatic 4T1 lung lesion growth *in vivo*.** (**A**) UMAP embedding of 104,219 cells from Mouse 3 Tumor 1 (M3 T1) Sections 1-3 (S1-S3) where each cell is colored by its assignment to 18 cell types annotated with this library. Cell types and colors are listed below the UMAP. (**B**) Spatial organization of cell types in M3 T1 S1-S3. Cell types colored using the annotations in (A). Scale bar = 1 mm. (**C**) Gene expression profiles for all 175 probed genes across the 18 cell types annotated with this library. Dot color indicates the mean gene expression for a marker gene within a cell type, while dot size indicates the percent of cells within a cell type that express that gene. (**D**) Pearson correlation between average gene expression of shared genes in the 124-gene MERFISH library for M1/M2 versus the 175-gene library for M3 for each cell type resolved by both libraries. (**E**) Pairwise local correlation of gene expression MERFISH counts between cancer cell Hotspot modules. (**F**) Heatmap of average cell type densities in each cancer cell Hotspot module. (**G**) Character matrix and phylogeny for the 10,061 cells with >60% intBC detection efficiency across S1 to S3 for M3 T1. Integration barcodes (intBCs) are listed below the character matrix, which is colored by lineage mark (LM) identity for each edit site; LM key shown to the right of the character matrix; UE = unedited, ND = not detected. Clades 1 through 18 are colored on the phylogeny with a key at the bottom. NA = not assigned. (**H**) Scatterplot of fitness versus percentage of edit sites with LM installed for tumors included in this study. (**I**) Distribution of percentage of sites edited across cancer cell Hotspot modules. (**J**) Pearson correlation with malignant cell boundary distance versus Pearson correlation with malignant cell fitness for cell type densities and gene expression (expr.) signatures. Dashed line depicts y = -x as a guide. Corr. = correlation. (**K**) Heritability versus spatial variation for M3 T1 gene expression quantified with Moran’s *I* autocorrelation. Dashed line depicts y = x as a guide.

(**L**) Spatial organization of malignant cell fitness (left), *Cldn4* MERFISH counts (middle), and *Fgfbp1* MERFISH counts in M1 T1. S3 shown as a representative section. Scale bar = 500 µm. Cell type abbreviations are as follows: Malig. = Malignant, Mac. = macrophage, Treg = regulatory T cell, cDC = conventional Dendritic Cell, Neu. = Neutrophil, NK = Natural Killer cell, Endo. = endothelial, CAF = Cancer Associated Fibroblast, AF1/AF2 = Alveolar Fibroblast Type 1/2, AT1/AT2 = alveolar epithelial type 1/2, CAP1/CAP2 Endo. = Capillary type 1/2 Endothelial. See table S24 for relevant statistics.


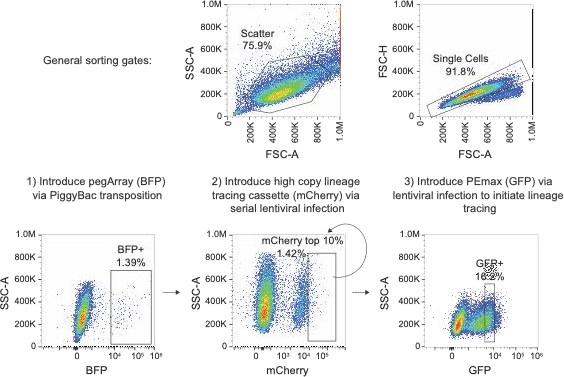


**Fig. S16. Representative flow cytometry sorting gates for cell line engineering with PEtracer components.** Cells were sorted based on expression of the indicated fluorescent markers, with percentage of cells from the parent gate indicated. For generation of cells with high numbers of integrated tracing cassettes, cells were serially infected with lineage tracing cassette lentiviral libraries and the brightest 10% of mCherry expressing cells were sorted to isolate cells with high integration numbers. This process was repeated to increase integration count until the average number of integrations in the population was over 10 as evaluated by qPCR.

### Tables S1-S30 (separate file)

S1 N states vs. entropy simulation

S2 N states vs. edit saturation simulation S3 Parameter sweep simulation

S4 Branches with edit simulation

S5 Predited crosshyb by insert length S6 Lineage cassette intBCs

S7 Insert screen logFC

S8 Insert predicted crosshyb S9 pegArray edit counts

S10 Edit rates for protospacer variants S11 Fully-edited clone whitelist

S12 intBC probe library S13 LM probe library

S14 Common sequence probe library S15 Readout probes

S16 Adaptor probe library

S17 Barcoding phylogeny statistics S18 Barcode edit sites downsampling

S19 Barcode detection rate downsampling S20 Colony phylogeny statistics

S21 124-gene MF probe library S22 Tumor imaging statistics S23 Tumor phylogeny statistics

S24 Predictors of phylogenetic fitness S25 175-gene MF probe library

S26 Heritability and spatial variation of gene expression and modules S27 Miscellaneous p-values

S28 TruSeq primer design sheet S29 Nextera primer design sheet S30 8-mer pegArray design guide

### Movie S1.

Time-lapse brightfield imaging of 4T1 cells growing on a glass coverslip over six days (Incucyte).
